# Multiple symmetric lipomatosis (Madelung's disease) with symptoms and signs of hypertension, lipodystrophy, and dyspnea: A case report and literature review

**DOI:** 10.1002/ccr3.4288

**Published:** 2021-06-24

**Authors:** Christos Tsilivigkos, Stylianos Mastronikolis, Spyridon Lygeros, Dimitra Tsilimpoti, Theodoros Papadas

**Affiliations:** ^1^ Department of Otorhinolaryngology‐Head and Neck Surgery General University Hospital of Patras Patras Greece; ^2^ Department of General Surgery General University Hospital of Patras Rion Greece; ^3^ Department of Plastic Surgery ‘Agios Andreas’ General Hospital of Patras Patras Greece

**Keywords:** case report, lipodystrophy, madelung's disease, multiple symmetric lipomatosis

## Abstract

Madelung's disease generally refers to a benign symmetrical lipomatosis of the neck, but its presentation can vary. It is treated surgically and different approaches can be implemented. In cases of a threatened airway, a tracheostomy can be performed.

## Graphical abstract

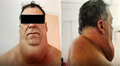

## 1 INTRODUCTION

Madelung's disease generally refers to a benign symmetrical lipomatosis of the neck, although its presentation can vary. The main treatment is surgical and different approaches can be implemented. In cases of a threatened airway, a tracheostomy can be performed.

The first to report benign symmetrical lipomatosis was Sir Benjamin Brodie in 1898,^1^ after the descriptions of Otto Wilhelm Madelung in 1888 and Launois and Bensaude in 1898.^2^, ^3^ The disease has an incidence of 1:25 000; it is usually described in patients of 30 to 60 years old and occurs more often in men than in women (15:1‐30:1).^4^

Patients with Madelung's disease were classified by Enzi into two categories based on the distribution of fatty masses: in type I, the fatty masses are distinct, symmetrical, protruding from the body surface, and giving the patient a “pseudoathletic” appearance, whereas in type II there is diffuse involvement of the subcutaneous tissue, giving the patient an obese appearance.^5^

Alcohol consumption, chiefly in the form of red wine, is recorded in most cases of Madelung's disease (60%‐90%). According to a study by Enzi et al, the daily ethanol intake for these patients was 148.4 ± 71.3 mL, ranging from 50 up to 400 mL per day. In this study, it was concluded that alcohol discontinuation leads to a slight regression of the disease, while an increase in alcohol intake accelerates fat growth. However, there were patients whose fat masses remained stable and even enlarged following alcohol abstinence.^6^

We report the case of a patient with Madelung's disease, our treatment plan, and the patient's postoperative course. The current case report is of clinical interest because of the massive lipomatosis, leading to a threatened airway during and after the operation, and because of the clinical decisions that we made during its management. We also conduct a thorough literature review.

## 2 CASE PRESENTATION

**FIGURE 1:**
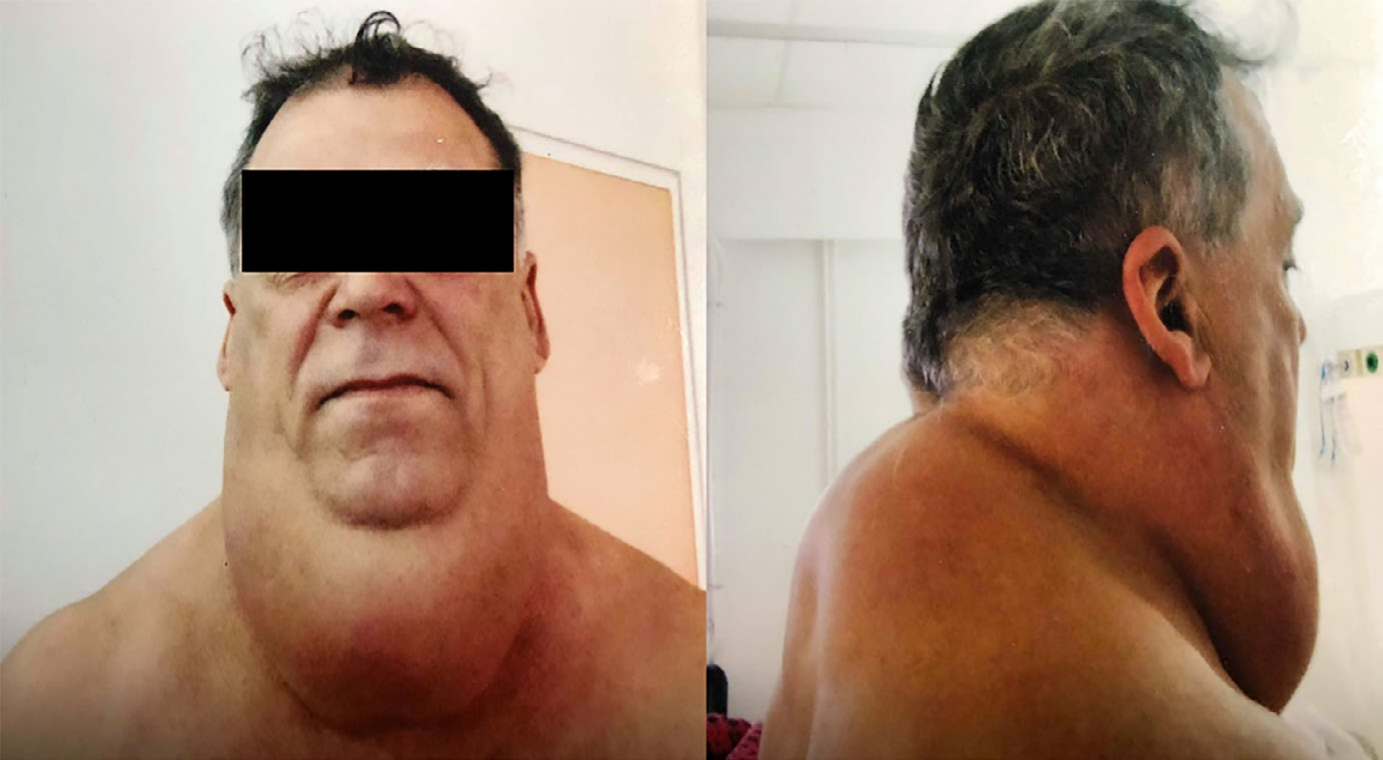
The patient upon presentation

A 56‐year‐old man weighing 102 kg and 188 cm tall (Figure 1) was referred to our hospital because of an evident thickening around the neck. He had had the enlargement for the last 10 years and lately, the condition worsened. He was also a heavy alcohol drinker for the last 30 years and had 50 pack‐years of smoking upon presentation. He was employed as a farmer and construction worker and had never demanded health care for his condition in the past.

More specifically, during the last 3 years, the enlargement became a collar‐like mass around the neck and dyspnea crises with cough and upper airway excretions appeared, especially when bending the neck. He was relieved after some minutes of neck extension. The patient reported progressive difficulty sleeping due to extensive bulk of the mass, while he never had a problem of snoring, sleep deprivation, or other sleep apnea‐associated symptoms. Additionally, he complained of weakness and chronic fatigue during these last years. The mass presented with cervical pain upon weight lifting, while he did not have any signs of neuropathy.

He referred a known history of hypertension with frequent hypertensive crises with systolic blood pressures of 220‐230 mm Hg after heavy drinking, for which he used an angiotensin II receptor blocker (ARB) and hydrochlorothiazide and was usually referred to the local hospital. The cardiac assessment was normal. He had suffered from hypothyroidism for the last 15 years.

Upon examination, he had a circular mass of the neck, semi‐hard on the anterior and hard on the posterior in a double oval‐shaped configuration. The mass was not fixed to the overlying skin. His articulation was disturbed, and he had stridor while bending the head. Interestingly enough, we noticed that his legs and arms had lipohypertrophic along with lipoatrophic areas and that his supraclavicular regions were hypertrophic. The identification of the “pseudoathletic” type of Madelung's disease (type I), and hence, the diagnosis was delayed because the patient's physique was attributed to his occupation as a construction worker. Neurological examination was normal.

High values of uric acid, cholesterol, triglycerides, CPK, and LDH were reported. A slight elevation of SGOT along with a double γ‐GT price was compatible with alcohol abuse.

**FIGURE 2:**
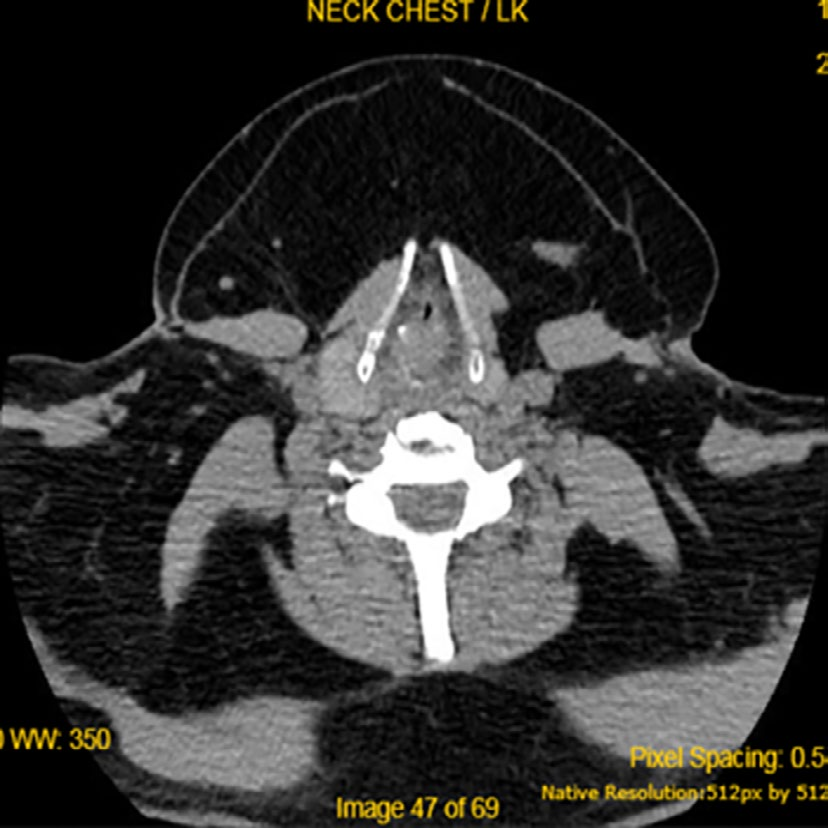
Diffuse lipomatosis on CT scan

A facial, cervical, and thoracic computed tomography (CT) showed a bilateral and symmetrical fat mass of the posterior and anterior region of the neck and of the upper trunk, without displacement or infiltration of the trachea (Figure 2). Based on these findings, the diagnosis of Madelung's disease was made and we decided to treat the patient surgically.

**FIGURE 3:**
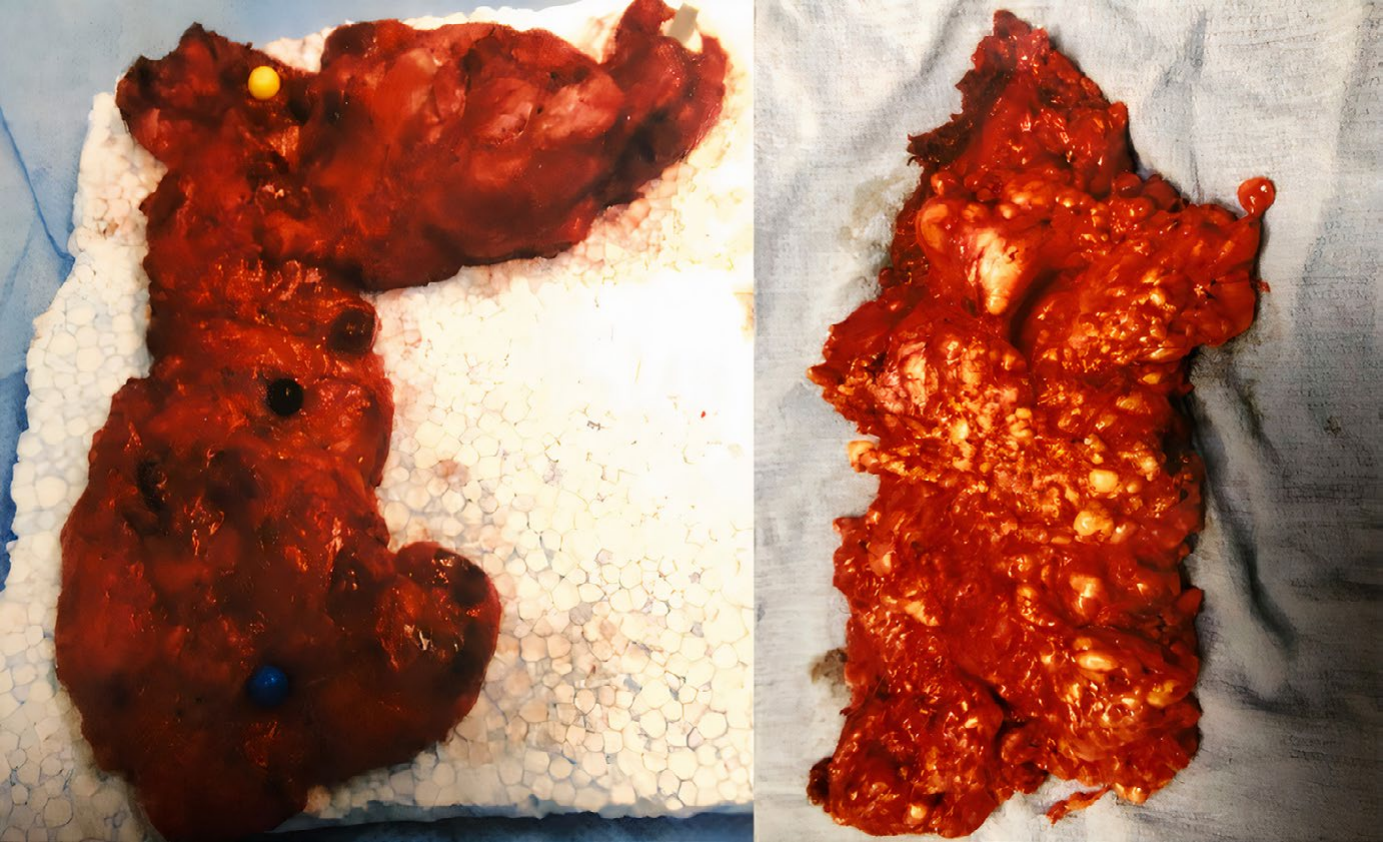
Anatomic bilateral dissection of the fat tissues from the anterior cervical region

Two different operations took place. In the first one, orotracheal intubation was impossible, so nasotracheal intubation was performed. A Gluck‐Sorenson incision was performed, and fat tissue was excised in a similar fashion as in bilateral functional lymph node neck dissection with preservation of the accessory and marginal and cervical branches of the facial nerves. More specifically, we dissected levels I‐V on the right side and levels I‐VI on the left side. The dissection of the tissue in the carotid triangles was laborious (the fat tissue had septa that extended between the structures). The excised specimen's size was 27 × 12 × 18 cm, and its weight was 627 g (Figure 3). A tracheostomy was performed before completion.

In the second operation, which took place 3 weeks later, the patient was placed in a prone position. The fat masses of the cervical, occipital, suboccipital, retro‐auricular, and upper dorsal regions were removed surgically and with liposuction. The weight of the fat tissue was 114gram. We continued with removing the fat mass from the upper anterior cervical region. Part of the skin was removed because of its excess. The tracheostomy from the first operation remained and was finally removed 6 days after the second operation.

Histological examination after both the operations confirmed the initial diagnosis.

During the next 6 months, we suggested the use of a soft collar because we desired extra tension of the musculocutaneous tissue over the neck triangles for better healing. The patient did not follow our orders for alcohol abstinence.

**FIGURE 4:**
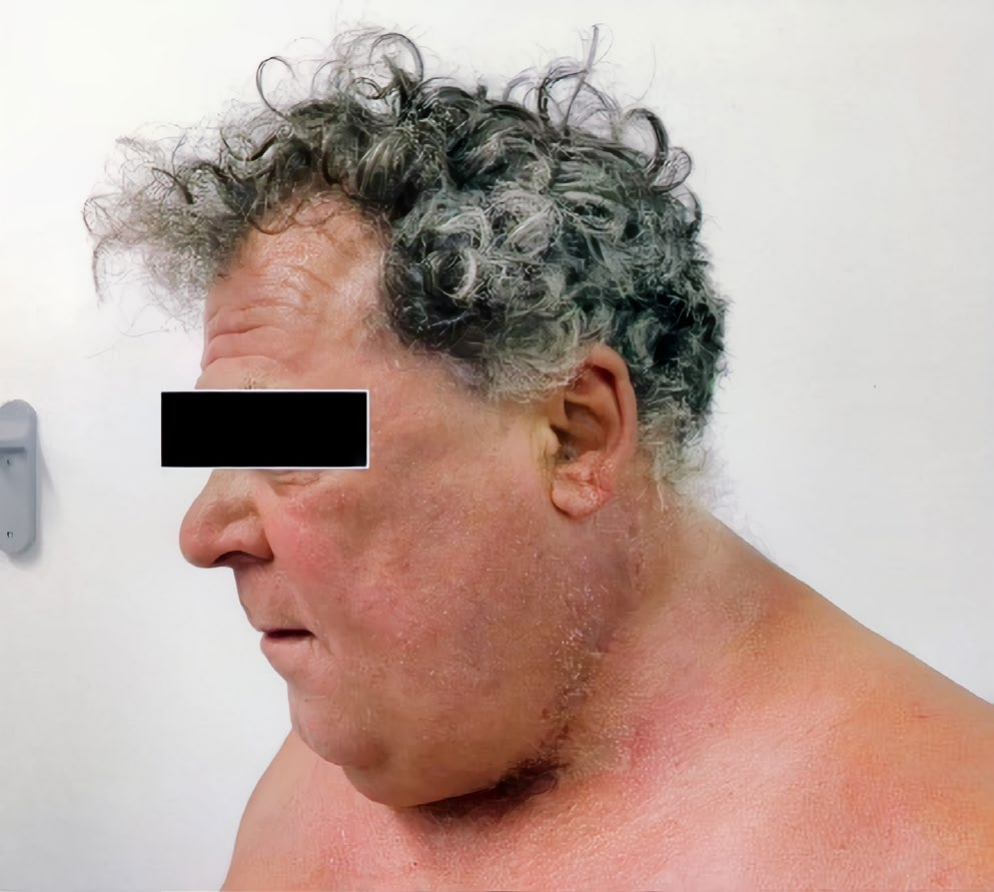
The patient 3 months after the last operation

In Figure 4, we can see the final result three months later.

## 3 DISCUSSION

Benign symmetrical lipomatosis is characterized by multiple, symmetric, disfiguring, and nonencapsulated fat proliferation in the head, neck, trunk, shoulders, and proximal arms. These distinguish Madelung's disease from lipomas, which usually are encapsulated and symmetric or asymmetric, and from lipodystrophy syndromes, which combine lipohypertrophy and lipoatrophy.^7^ It is a benign disease with very rare references to malignant transformation.^8^, ^9^

The cause of Madelung's disease is unknown. The disorder is generally acquired, but there are also familial cases with an autosomal dominant pattern of inheritance.^10^

Mitochondrial disorders are observed in some patients: reduced cytochrome c oxidase activity and functional deficiency in the activity of respiratory chain complex IV, ragged red fibers and multiple deletions of mitochondrial DNA, and rarely a single large‐scale mitochondrial DNA deletion.^11^, ^12^, ^13^ In addition, patients with myoclonic epilepsy with ragged red fibers (MERRF) syndrome and the A‐>G transition at nucleotide 8344 of the mitochondrial DNA presented with Madelung's disease. Another point mutation associated with the disorder is m.8363A>G mtDNA mutation.^14^, ^15^

Moreover, Madelung's disease is most probably a mitochondrial disorder of brown fat, as the expression of UCP‐1 mRNA in resected tissue was proven.^16^ Histological examination indicates the brown fat tissue origin, as multivacuolar fat droplets and the presence of mitochondria with abundant and large cristae are observed.^10^ Enzi et al (1977) suggested a block in catecholamine‐stimulated lipolysis in fat tissue of patients with Madelung's disease at a step preceding the formation of cAMP.^17^

According to previous literature, multiple symmetric lipomatosis is probably a symptom of liver disease, since the latter preexists in most cases.^18^ Alcohol has been shown to up‐regulate the expression of the lipin‐1 gene through inhibition of AMPK and activation of SREBP‐1.^19^ In another pathway, miRNA‐217 promotes ethanol‐induced fat accumulation through down‐regulating SIRT1.^20^ These two pathways are indicative of the pathology of alcoholic liver disease and most probably of the pathology of Madelung's disease.

The diagnosis of Madelung's disease is made clinically and with the assistance of computed tomography (CT) and magnetic resonance imaging (MRI), while sonography is used but is less helpful.^21^ These methods also help us differentiate lipomatosis from liposarcoma, even if clinical examination, because of the characteristic Madelung's collar, leads to diagnosis in most of the cases. The so‐called “Madelung's collar” grows slowly over several years, but there are cases where enlargement occurred in less than a year. The lipomatosis texture can be from soft to hard.

The clinical presentation of the disorder can be described with the assistance of a newer classification as shown in Table 1.^22^

**TABLE 1:** Classification for Madelung's disease^22^

Type	Clinical presentation
I	Madelung's collar (localized form)
II	Pseudoathletic type: shoulder girdle, deltoid region, arms, torso
III	Gynecoid type: pelvic girdle
IV	Abdominal type: abdomen

The disorder can rarely be found in the form of lipomatosis of the tongue, which presents as macroglossia and which is treated surgically with partial glossectomy.^23^

According to Enzi et al, there is a mortality rate of 17.8 deaths/1000 patients/year and the most frequently reported cause of death is that of sudden death. An impairment of cardiovascular autonomic reflexes may be a poor prognostic factor in Madelung's disease, as in diabetes mellitus. Very interestingly, an absence of coronary disease is reported in these patients. The metabolic profile of the disorder includes increased HDL cholesterol, decreased LDL cholesterol, increased triglycerides, impaired glucose tolerance, and high frequency of hyperuricemia and renal tubular acidosis.^7^

Compression and displacement of important structures are common with symptoms of dyspnea, stridor and cough, obstructive sleep apnea, dysphagia, mediastinal syndrome, and superior vena cava syndrome.^7^

Neuropathy constitutes an important factor of morbidity in these patients. It seems like it is not alcohol‐induced but a chronic distal axonopathy is an integral part of the syndrome.^24^ Histologically, it is characterized by nerve fiber loss, affecting large myelinated fibers in contrast to alcoholic neuropathy in which we see demyelination and axonal degeneration.^25^ It consists of motor and sensory neuropathy which present with muscular cramps and weakness and with paraesthesias, respectively. Autonomic neuropathy has minor presentations such as hyperhidrosis and major ones, such as sudden cardiac death.^7^ Mitochondrial disorders could be the cause of this neuropathy.

Other symptoms include alcoholic fatty liver, glucose intolerance and diabetes mellitus, hypothyroidism, macrocytic anemia, hypertension, and generalized weakness.^10^, ^26^

At this point, we would like to examine the indications for treatment and our options. These patients are treated when complications, such as neuropathy, diabetes, trachea displacement, mediastinal syndrome, superior vena cava syndrome, and hypertension, occur and also for aesthetic and psychological reasons. Dyspnea and stridor improved significantly after surgery in our case. Serious hypertensive episodes with SBP of 220‐230 mm Hg that appeared before and that required pharmacological treatment and short hospitalization ceased to exist. Following surgery, he was normotensive and had no need for drug therapy, even after alcohol abuse. The generalized weakness that he experienced no longer existed after surgery, and he returned to work. Although his sleep normalized, the patient complained of snoring, which he had never experienced before. This could possibly be because of his sleeping in an upright position over the past few years.

As already discussed, alcohol abstinence can be beneficial. Fischer et al proposed the intralesional injection of enoxaparin but further investigations are required.^27^ Leung et al proposed the use of oral salbutamol, a β2‐agonist, which could possibly stabilize the fat mass. The use of a β2‐agonist could be considered in the case of multiple fat masses that we cannot excise.^28^

The classical treatment of the disorder is cold steel surgery. Open lipectomy and dermatolipectomy allow resection of larger fat debulking with safe control of the facial nerve and the vasculature. A long mid‐neck transverse skin incision is superior to multiple local incisions according to Wong et al^29^ Complications such as seromas, hematomas, infections, lymphatic fistulas, and the formation of scars are common. Reduced range of motion of the head is very common after surgery, and fat relapse is not uncommon.^30^ Tumescent liposuction is another treatment option.^26^

We combined both open surgery and liposuction. Our treatment reasoning was that we should first debulk the anterior massive fat. During the procedure, we realized a secondary chondromalacia of the trachea and a threatened airway, due to chronic compression from lipomatosis. That is why we decided on a second operation 3 weeks later for the posterior fat mass. A tracheostomy that was performed during the first operation remained during the whole procedure securing the airway. Liposuction was only used in the second operation because the posterior part of the neck lacks noble structures, such as large vessels and the accessory nerve. This way, a severe complication is much less possible.

Finally, we believe that the definitive solution to the difficult treatment of Madelung's disease will be given when the pathophysiology of the disorder will be clarified, so that specific drug targets will be highlighted.

## CONFLICT OF INTEREST

None declared.

## AUTHOR CONTRIBUTIONS

CT: involved in conception and design of the work, photography, review of the literature, main contribution to writing, and final approval. SM: involved in conception and design of the work, contribution to writing, review of the literature, and final approval. SL: involved in conception and design, drafting the article, and final approval. DT: involved in drafting the article and final approval. TP: served as academic advisor, revising it critically for important intellectual content and provided final approval.

## ETHICAL APPROVAL

Written informed consent was obtained from the patient.

## Data Availability

All data underlying the results are available as part of the article, and no additional source data are required.
